# Genotype and phenotype analysis and transplantation strategy in children with kidney failure caused by NPHP

**DOI:** 10.1007/s00467-022-05763-3

**Published:** 2022-10-13

**Authors:** Jianyi Li, Xiaojun Su, Huanxi Zhang, Wenrui Wu, Jianming Li, Yanxu Chen, Jun Li, Qian Fu, Chenglin Wu, Xuhui Zhong, Changxi Wang, Longshan Liu

**Affiliations:** 1 Organ Transplant Center, The First Affiliated Hospital, Sun Yat-Sen University, No. 58, Zhongshan Second Road, Yuexiu District, Guangzhou, 510080 China; 2 Department of Pediatrics, Peking University First Hospital, No. 1, Xi An Men Da Jie, Xi Cheng District, Beijing, 100034 China; 3 Guangdong Provincial Key Laboratory of Organ Donation and Transplant Immunology, Guangzhou, 510080 China; 4 Guangdong Provincial International Cooperation Base of Science and Technology (Organ Transplantation), Guangzhou, 510080 China

**Keywords:** Children, Kidney transplantation, Nephronophthisis, Boichis syndrome

## Abstract

**Background:**

Nephronophthisis-related ciliopathies (NPHP-RC) have strong genotype and phenotype heterogeneity, and the transplantation strategy of Boichis syndrome is still controversial. Our purpose was to examine associations of genotype and phenotype in children with NPHP-RC and analyze the transplantation strategies of different phenotypes.

**Methods:**

The records of children with NPHP treated at our center from 01/2018 to 03/2021 were retrospectively reviewed. Inclusion criteria were a diagnosis of NPHP, received kidney transplantation, and received whole exome sequencing (WES) or nephropathy gene panel testing.

**Results:**

Twenty-nine children with NPHP were included. Nine children (31%) had *NPHP1* mutations, and all presented with isolated nephropathy. Eighteen of 20 patients with non-*NPHP1* mutations had compound heterozygous mutations, and 70% had extrarenal phenotype. Age at disease presentation (11.2 ± 1.94 years) and the development of kidney failure (12.4 ± 2.70 years) were later in children with *NPHP1* mutations than those with non-*NPHP1* mutations (5.2 ± 2.83 years and 5.7 ± 2.92 years, respectively). Four of six children with *NPHP3* mutations were diagnosed with Boichis syndrome due to liver fibrosis. Isolated kidney transplantation resulted in good outcomes for patients with mild or moderate liver fibrosis without portal hypertension, while cholestasis was common postoperatively and could be resolved with ursodeoxycholic acid.

**Conclusions:**

*NPHP1* mutations are the most common in children with NPHP, and the phenotype of *NPHP1* mutation is significantly different from that of *non-NPHP1* mutation. For NPHP patients with mild to moderate liver fibrosis without portal hypertension, timely treatment of cholestasis could prevent the rapid progression of liver function damage after isolated kidney transplantation.

**Graphical abstract:**

**Figure:**
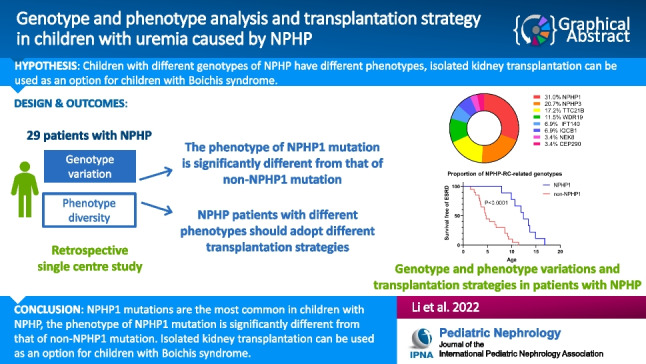
A higher resolution version of the Graphical abstract is available as Supplementary information

**Supplementary Information:**

The online version contains supplementary material available at 10.1007/s00467-022-05763-3.

## Introduction

Nephronophthisis-related ciliopathies (NPHP-RC) are autosomal recessive disorders characterized by kidney corticomedullary cysts and extrarenal symptoms and are the most common genetic cause of kidney failure in children and young adults [1–3]. NPHP-RC can present as isolated nephropathy or as a syndrome [4]. The incidence of extrarenal involvement in patients with NPHP-RC ranges from 10 to 50%, and the most commonly involved sites are the liver, eyes, bones, and central nervous system (CNS) [5–8]. Currently, variants of around 90 genes have been identified as causes of NPHP-RC [9, 10].

Most patients with NPHP-RC progress to kidney failure before the age of 30 years [8, 11]. Typically, patients with juvenile nephronophthisis initially present with polydipsia, polyuria, secondary enuresis, growth retardation, and anemia. As such, diagnosis is usually delayed, and patients are diagnosed at a later disease stage [12]. The development of next-generation gene sequencing technology (NGS) has resulted in genotyping of a larger number of patients with NPHP-RC, leading to studies of the relationship between genotype and phenotype [7, 8, 13, 14].

In the aforementioned studies, most patients were in the chronic kidney disease (CKD) phase of the disease at the time of phenotype analysis, or only some patients had reached kidney failure during the follow-up period. As nephropathy progresses to the kidney failure stage, the involvement of extrarenal organs also progresses. Thus, the analysis of the phenotype of uremic patients is helpful for the perioperative management of kidney or other organ transplantation. For example, when there is liver involvement, the state of liver function plays a decisive role in perioperative management and the choice of transplantation strategy.

When patients with NPHP-RC reach kidney failure, kidney transplantation is the first-choice treatment [15]. Some studies have shown that compared with the general pediatric kidney transplant population, the effect of kidney transplantation is better in recipients with NPHP-RC as the primary disease [16]. Boichis syndrome is a unique NPHP-RC that is characterized by congenital NPHP and congenital hepatic fibrosis [17]. Treatments for this disease include combined liver and kidney transplantation (CLKT), sequential liver and kidney transplantation, and isolated kidney transplantation [18, 19]. In patients with Boichis syndrome, portal hypertension can lead to adverse events due to the rapid progression of hepatic fibrosis after kidney transplantation only [20, 21]. Generally, the transplantation strategy is adopted mainly based on the degree of liver fibrosis. When the degree of liver fibrosis meets the conditions for liver transplantation, it is necessary to perform CLKT. However, there is no clear guidance on the optimal surgical strategy for patients with mild to moderate liver fibrosis (especially those with high-risk genotypes). CLKT can solve both liver fibrosis and kidney failure, yet the surgical risk is high. The surgical success rate of isolated kidney transplantation is high; however, it is uncertain when liver fibrosis progresses to the indication of liver transplantation. Thus, the purpose of this study was to examine the association between NPHP genotype and phenotype in children to provide references for clinicians when determining an appropriate transplantation strategy.

## Methods

### Study design and participants

The genotype and phenotype of children with NPHP-RC treated at our center from January 1, 2018, to March 31, 2021, were retrospectively reviewed. Inclusion criteria were a diagnosis of NPHP, received a kidney transplant or CLKT, received whole exome sequencing (WES) or nephropathy gene panel testing and were found to have a pathogenic gene mutation associated with NPHP, had complete medical records, and were ≤ 18 years of age. Diagnosis of NPHP was based on the clinical diagnostic criteria for NPHP proposed by Chaki et al. [8]. Exclusion criteria were (1) WES or nephropathy gene panel was not performed; (2) NPHP-related pathogenic mutation was not present; and (3) the patient did not meet the clinical diagnostic criteria of NPHP-RC.

The study was approved by the Ethics Committee of the First Affiliated Hospital of Sun Yat-sen University (IIT-2021–892).

### Data collection

Data extracted from the medical records included medical history, including age at presentation of NPHP-RC and age when kidney failure was reached. Laboratory data extracted included serum creatinine (Scr), blood urea nitrogen (BUN), albumin (ALB), globulin (GLB), alanine aminotransferase (ALT), aspartate aminotransferase (AST), and total bilirubin (TB) levels. All children underwent abdominal ultrasound. A liver biopsy was performed if abdominal ultrasound indicated liver fibrosis when the informed consent was available. All the children underwent chest, extremity, and pelvis X-rays. All children received visual acuity and visual field evaluation, slit lamp examination if necessary, and optical coherence tomography and electrophysiological examinations in cooperative patients suspected of having retinitis pigmentosa. Each child underwent an echocardiogram to check for structural defects in the heart. Head MRI examinations were performed in children with neurological abnormalities found by physical examination to detect cerebellar vermis hypoplasia and other structural brain abnormalities. All patients were followed up through outpatient clinic and telephone after kidney transplantation. Postoperative eGFR, graft survival, patient survival, rejection, and recurrence of primary disease were obtained by follow-up.

### Gene sequencing and data analysis

WES was performed using MyGenetics and Wuxi NextCODE. Genomic DNA was extracted from blood lymphocytes. Exon capture was performed using Agilent SureSelect Human all Exome V5 Kit, NimbleGen, or MyGenostics Gencap Capture techniques, and then NGS was performed on the Illumina HighSeq sequencing platform. At least 98% of the target sequences were sequenced at a 20 × reading depth.

After removing reads containing adaptor sequences and low-quality reads, clean data were mapped to the human reference genome assembly (NCBI build 37/hg19) using the Burrow–Wheeler Aligner (BWA). Single nucleotide polymorphisms (SNP) and small insertions and deletions (INDELs) were detected using a Genome Analysis Toolkit (GATK). Allele frequency was determined by annotating the variations using public databases (genomAD, Human Genome Mutation Database (HGMD), and the 1000 Genomes Project). Changes that represented synonymous and common variants (minor allele frequency > 1%) were discarded. SNP variant deleteriousness was predicted by SIFT, PolyPhen2, and MutationTaster. Mutation screening was prioritized based on known disease-causing genes and nephropathy-associated genes. Evidence of disease causality was assessed using ClinVar and HGMD, followed by a manual review of the cited primary literature. Copy number variation (CNV) analysis was performed using WES data, and then validated by PCR. Using the *ALB* gene as the internal reference gene, the copy number of exon 1–20 of *NPHP1* was detected by fluorescence quantitative PCR with normal control samples and proband and family samples.

The nephropathy gene panel includes 162 genes causative or associated with nephropathy, as well as genes that may cause phenocopies in humans or related phenotypes in animal models (Supplementary Table 1). A custom NimbleGen SeqCap EZ Choice Library (NimbleGen; Roche, Madison, WI) was used to capture all exons and exon–intron boundaries (plus 50 base pairs at each end) of these genes for a final targeted region of 1.05 Mb.

Pathogenicity assessment was conducted by a team of nephrologists and molecular geneticists. According to American College of Medical Genetics (ACMG) guidelines, mutations in known pathogenic genes whose pathogenicity levels are “pathogenic,” “likely pathogenic,” and “variant of unknown significance (VUS)” are defined as diagnostic variants. For variants of unknown significance, we regarded it as a diagnostic variation only when other causes of chronic kidney failure were excluded and there was a high correlation between phenotype and variants. All diagnostic variants were confirmed by Sanger sequencing with segregation.

### Statistical analysis

Continuous data were expressed as mean ± standard deviation or median and interquartile range (IQR), and categorical data as count (percentage). Comparisons of patients with and without *NPHP1* mutations were performed using the *t*-test. Kidney survival analysis between children with *NPHP1* mutations and those with non-*NPHP1* mutations was performed by Kaplan–Meier analysis. The level of statistical significance was defined as *p* < 0.05. GraphPad Prism 8 statistical software was used for all statistical analyses.

## Results

### Patient selection and clinical data

**Fig. 1:**
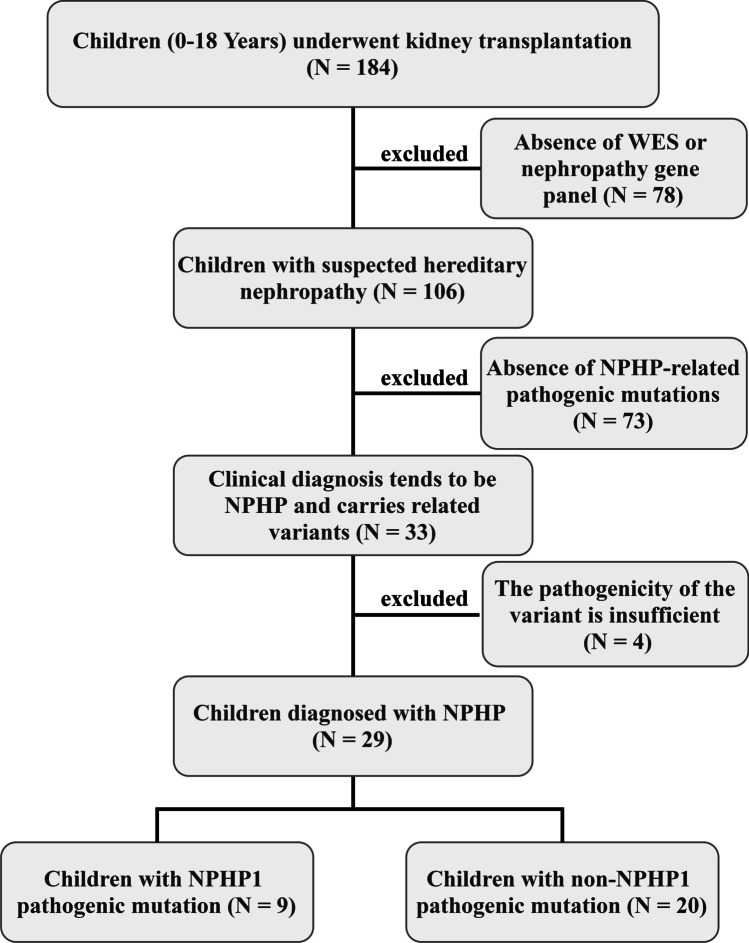
Flow diagram of patient selection. WES, whole exome sequencing

A flow diagram of patient inclusion is shown in Fig. 1. The records of 184 children and adolescents who received kidney transplantation at our center were identified and reviewed from January 1, 2018, to March 31, 2021. Of the 184 patients, 106 received WES or nephropathy gene panel analysis due to suspected hereditary nephropathy; therefore, the 78 patients who did not receive these tests were excluded. The clinical diagnosis of 33 children tended to NPHP and carried variants in genes related to NPHP, but four children were excluded because they carried monoallelic variants. Twenty-nine children (17 boys, 12 girls) were eventually included in the analysis.

The clinical data of the 29 children is summarized in Table 1. The mean age of NPHP presentation was 7.1 ± 3.81 years, and the mean age of reaching kidney failure was 7.8 ± 4.23 years. The median follow-up time was 21 months (*IQR*: 10–27 months). The median eGFR value post-transplant was 100 ml/min/1.73 m^2^ (*IQR*: 91–112). One child underwent CLKT, and the others received kidney transplantation only. Graft loss occurred in three patients due to acute thrombosis or acute kidney vein torsion. All three patients received second transplantation; two grafts survived well after the second transplant, and in one case, there was secondary acute thrombosis. There was no rejection or recurrence of primary disease, and there was no recipient death.

**Table 1:** Patient clinical features

Parameter		Patients with *NPHP1* mutations (*n* = 9)	Patients with non-*NPHP1* mutations (*n* = 20)	Total (*n* = 29)	*P*
Sex (male)		6 (66.7%)	11 (55%)	17 (58.6%)	0.649
Age of disease presentation, years		11.2 ± 1.83	5.2 ± 2.76	7.1 ± 3.81	< 0.001
Age of kidney failure, years		12.4 ± 0.58	5.7 ± 2.85	7.8 ± 4.23	< 0.001
Extrarenal manifestations		0 (0%)	14 (70%)	14 (48.3%)	< 0.001
Transplant age		14 (11.5, 15.5)	5 (4.5, 10)	9 (5, 11.5)	< 0.001
Follow-up time, months		24 (15, 27)	19 (8, 26)	21 (10, 27)	0.445
eGFR, ml/min/1.73 m^2^		116 (93, 154)	93 (89, 107)	100 (91, 112)	0.603
Transplant type	DD	9 (100%)	20 (100%)	29 (100%)	1
	LD	0 (0%)	0 (0%)	0 (0%)	1
Recurrence of primary kidney disease		0 (0%)	0 (0%)	0 (0%)	1
Rejection		0 (0%)	0 (0%)	0 (0%)	1
Graft failure		0 (0%)	4^a^ (16.7%)	4^a^ (12.1%)	0.532
Patient death		0 (0%)	0 (0%)	0 (0%)	1

Data presented as count, mean ± standard deviation, or median (interquartile range (IQR)). DD, deceased donor; *LD*, living donor. ^a^Three children with non-*NPHP1* mutations underwent a second kidney transplantation; one had graft loss after the second transplantation

### Genotype variation in patients with NPHP-RC

**Fig. 2:**
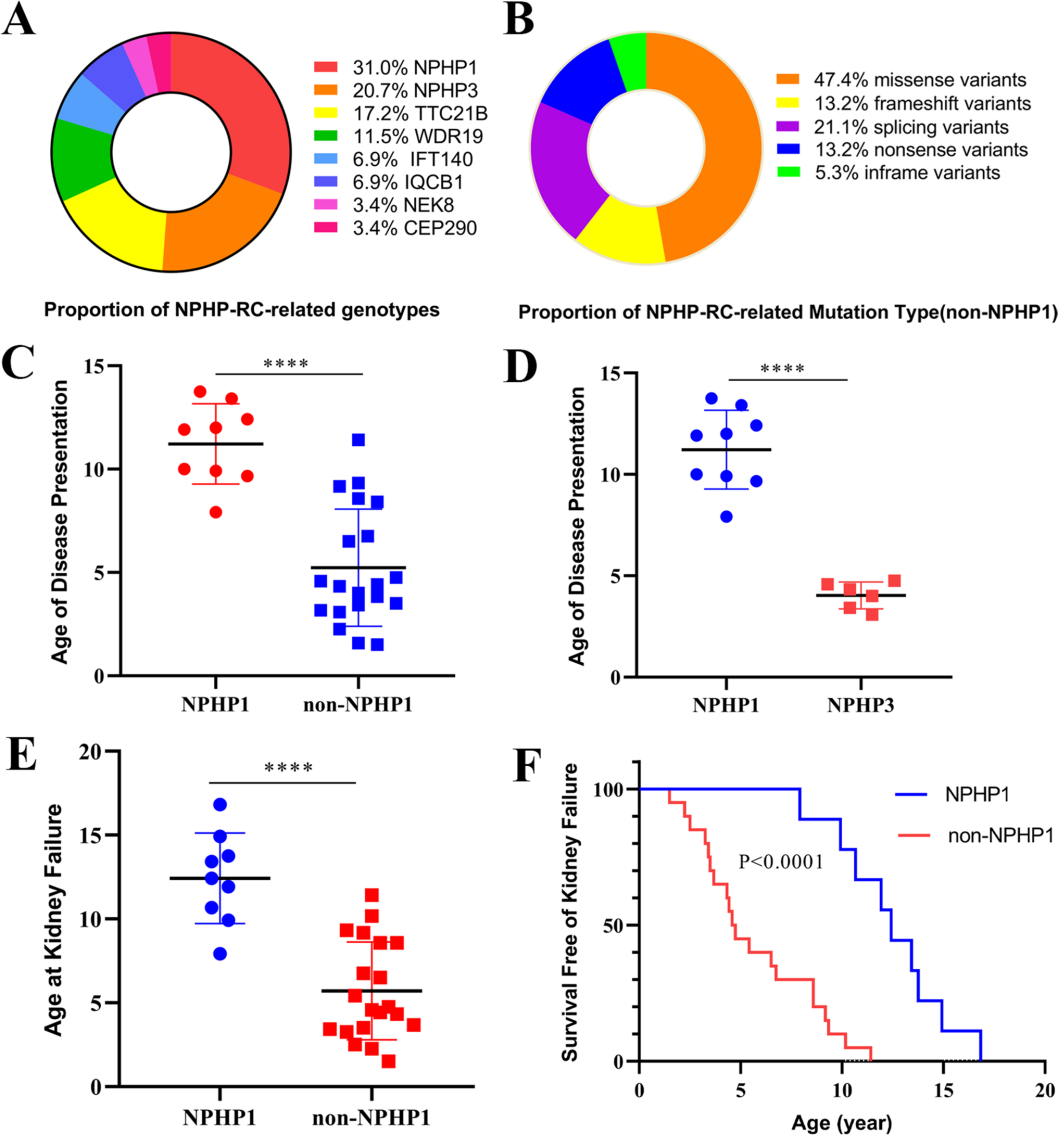
Genetic variations and the difference of phenotype in patients with NPHP. **A** Proportion of NPHP-RC-related genotypes. **B** Proportion of NPHP-RC-related mutation types (non-*NPHP1* mutation group). **C** and **D** Age of presentation in children with NPHP with different pathogenic mutations. **E** The age of reaching kidney failure in children with NPHP with different pathogenic mutations. **F** Kaplan–Meier analysis of kidney survival difference between children with *NPHP1* mutations (*n* = 9) and those with non-*NPHP1* mutations (*n* = 20)

In all patients, mutations in *NPHP1* (*n* = 9, 31%) were the most common genetic defects. Non-*NPHP1* mutations included mutations in *NPHP3* (*n* = 6, 20.7%), *TTC21B* (*n* = 5, 17.2%), *WDR19* (*n* = 3), *IFT140* (*n* = 2), *IQCB1* (*n* = 2), *NEK8* (*n* = 1), and *CEP290* (*n* = 1) (Fig. 2A and Table 2).

**Table 2:** Genotype–phenotype correlations in 20 children with non-*NPHP1* variants

Patient	Mutant gene	Nucleotide change	Amino acid change	Age of disease presentation	Age of kidney failure	Liver	Eye	Bone
1	*WDR19*	c.2963A > C	p.Gln988Pro	3 y, 2 mo	3 y, 3 mo	Yes		
	*WDR19*	c.3967-3974del	p.Ala1324Glufs					
2	*NPHP3*	c.3697-2A > C	splice site	4 y, 9 mo	5 y, 5 mo	Yes		
	*NPHP3*	c.1181 T > G	p.Ile394Ser					
3	*TTC21B*	c.T1552C	p.Cys518Arg	1 y, 7 mo	2 y, 6 mo			Yes
	*TTC21B*	c.530delA	p.Asp177fs					
4	*ICQB1*	c.1090C > T	p. Arg 364*	6 y, 9 mo	6 y, 9 mo			
5	*NPHP3*	c.2694-2_2694-1delAG	splice site	4 y, 4 mo	4 y, 4 mo	Yes		
	*NPHP3*	c.1304_1306delAAG	p.435del					
6	*TTC21B*	c.2323-2A > G	splice site	11 y, 5 mo	11 y, 5 mo			
	*TTC21B*	c.379G > A	p.Ala127Thr					
7	*NPHP3*	c.3813-3A > G	splice site	3 y, 5 mo	3 y, 5 mo	Yes		
8	*WDR19*	c.979 T > C	p.Trp327 Arg	8 y, 5 mo	8 y, 7 mo	Yes		
	*WDR19*	c.3703G > A	p.Glu1235Lys					
9	*CEP290*	c.2484-2A > G	splice site	6 y, 6 mo	6 y, 6 mo		Yes	
	*CEP290*	c.2586 + 1G > A	splice site					
10	*NEK8*	c.2018G > A	p.Cys673Tyr	2 y, 3 mo	2 y, 3 mo			
	*NEK8*	c.940G > A	p.Val314Met					
11	*NPHP3*	c.3813-3A > G	splice site	3 y, 1mo	3 y, 8 mo	Yes		Yes
	*NPHP3*	c.1135 T > C	p.Cys379 Arg					
12	*NPHP3*	c.1817G > A	p.Trp606*	4 y, 7 mo	4 y, 9 mo	Yes		
	*NPHP3*	c.1304_1306del	p.435del					
13	*IFT140*	c.745A > C	p.Thr249Pro	8 y, 7 mo	8 y, 7 mo		Yes	
	*IFT140*	c.3141G > A	p.Lys1047 Lys					
14	*TTC21B*	c.166G > A	p.Ala56Thr	3 y, 6 mo	3 y, 6 mo			
	*TTC21B*	c.1656_1659del	p.Cys552fs					
15	*TTC21B*	c.264_267dupTAGA	p.Glu90*	4 y, 5 mo	4 y, 5 mo	Yes		
	*TTC21B*	c.380C > T	p.Ala127Val					
16	*IQCB1*	c.1504C > T	p.Arg502*	9 y, 2 mo	9 y, 2 mo		Yes	
	*IQCB1*	c.1342_1343insC	p.Gln448fs					
17	*WDR19*	c.641 T > A	p.Leu214*	1 y, 6 mo	1 y, 6 mo	Yes		
	*WDR19*	c.2579C > A	p.Ala860Asp					
18	*IFT140*	c.3712G > A	p.Ala1238Thr	9 y, 4 mo	9 y, 4 mo			
	*IFT140*	c.2101G > A	p.Glu701Lys					
19	*TTC21B*	c.1656-1659del	p.Cys552fs	3 y, 10 mo	4 y, 7 mo	Yes		Yes
	*TTC21B*	c.1552 T > C	P.Cys518 Arg					
20	*NPHP3*	c.2132A > G	p.Asn711Ser	4 y	9 y, 2 mo			
	*NPHP3*	c.1082C > G	p.Ser361Cys					

With respect to mutation types, *NPHP1* mutations are quite different from non-*NPHP1* mutations. Of the 9 children who carried *NPHP1* pathogenic mutations, 8 had homozygous full-gene deletions of *NPHP1*. One patient with an *NPHP1* pathogenic mutation had a 1 allele point mutation, which led to a frameshift mutation, and the other allele had a large fragment deletion. Of all the non-*NPHP1* mutations, only two patients had homozygous mutations, and the other 18 had compound heterozygous mutations. A total of 38 pathogenic mutations were found in the non-*NPHP1* mutation group, including 5 nonsense variants (13.2%), 5 frameshift variants (13.2%), 2 inframe variants (5.3%), 8 splicing variants (21.1%), and 18 missense variants (47.4%) (Fig. 2B). Of all the variants, we found 15 novel variants that had not been previously reported (Supplemental Table 2). All alleles, segregation in the parents, and ACMG classification are shown in Supplemental Table 2.

### Phenotype diversity in patients with NPHP-RC

Overall, 15 of the 29 patients (51.7%) presented with isolated NPHP, while the other 14 (48.3%) presented with an extrarenal phenotype. The most common sites of extrarenal involvement were the liver, eyes, and bones. All patients with *NPHP1* mutations presented with isolated NPHP (Table 3), while 50% of patients with non-*NPHP1* mutations presented with hepatic involvement, 15% with ocular involvement, and 15% with skeletal involvement (Table 2). Liver involvement included elevated liver enzymes, hepatosplenomegaly, enlarged hepatic parenchyma echo, liver fibrosis, and portal hypertension; eye involvement included visual impairment and congenital bilateral optic nerve atrophy; skeletal involvement included abnormal shaping of the phalanx, metacarpal bone, tibia and fibula, uneven femoral bone density, and severe decrease of radius strength. Children with pathogenic mutations of *NPHP3* and *WDR19* were particularly susceptible to liver involvement (83.3% and 100%, respectively) (Table 3). Eye involvement was seen in patients with pathogenic *IFT140* (1/2), *IQCB1* (1/2), and *CEP290* (1/1) mutations (Table 3). Bone involvement was present in two of five patients with a pathogenic *TTC21B* mutation and one of six children with a pathogenic *NPHP3* mutation (Table 3).

**Table 3:** Genotype–phenotype correlations of 29 children

Mutant gene	Isolated NPHP	Age at kidney failure (y)	Liver involvement	Eye involvement	Bone involvement
*NPHP1* (*n* = 9)	100%	12.42 ± 0.58	-	-	-
*NPHP3* (*n* = 6)	16.7%	5.3 ± 2.28	83.3%	-	16.7%
*TTC21B* (*n* = 5)	40%	5.28 ± 3.16	40%	-	40%
*WDR19* (*n* = 3)	-	4.44 ± 3	100%	-	-
*IFT140* (*n* = 2)	50%	8.96 ± 0.38	-	50%	-
*IQCB1* (*n* = 2)	50%	7.96 ± 1.21	-	50%	-
*NEK8* (*n* = 1)	100%	2.25	-	-	-
*CEP290* (*n* = 1)	-	6.5	-	100%	-

Age at kidney failure presented as mean ± standard deviation

### Relationships between genotypes and age of disease presentation and kidney failure

Analysis of the age of disease presentation showed that the disease manifested earlier in children with non-*NPHP1* pathogenic mutations than in those with *NPHP1* pathogenic mutations (Fig. 2C). The mean age of disease presentation was 11.22 years in patients with *NPHP1* mutations and 5.23 years in those with non-*NPHP1* mutations. Of note, the age of disease presentation was the earliest (mean 4.03 years) in patients with *NPHP3* mutations (Fig. 2D).

Analysis of the age of reaching kidney failure showed that patients with non-*NPHP1* pathogenic mutations reached kidney failure significantly earlier than those with *NPHP1* pathogenic mutation (5.7 years vs. 12.42 years, *p* < 0.05) (Fig. 2E and F).

### NPHP3 mutations were associated with more severe phenotypes, leading to a higher incidence of Boichis syndrome

The presentation of disease symptoms was earliest in patients with *NPHP3* mutations, and liver involvement was present in five of the six children with *NPHP3* mutations. Three children underwent a liver biopsy, and a pathological examination of the tissue specimen showed mild to moderate fibrosis in all three children. Portal hypertension was present in one of the six children, and one had only mild liver enzyme elevations.

Of the six children with *NPHP3* pathogenic mutations, four (66.7%) were diagnosed with Boichis syndrome, and these were the only patients with Boichis syndrome of the 29 patients. Of the six children with *NPHP3* pathogenic mutations, five patients (5/6) with splice site mutations, truncation mutations, or frameshift mutations had liver involvement and rapidly progressed to kidney failure (mean age of reaching CKD stage 5: 4.3 years), while the patient (1/6) with only missense mutations had no extrarenal involvement and slow nephropathy progression (age of reaching CKD stage 5: 10.2 years).

### Transplantation strategy for Boichis syndrome

**Fig. 3:**
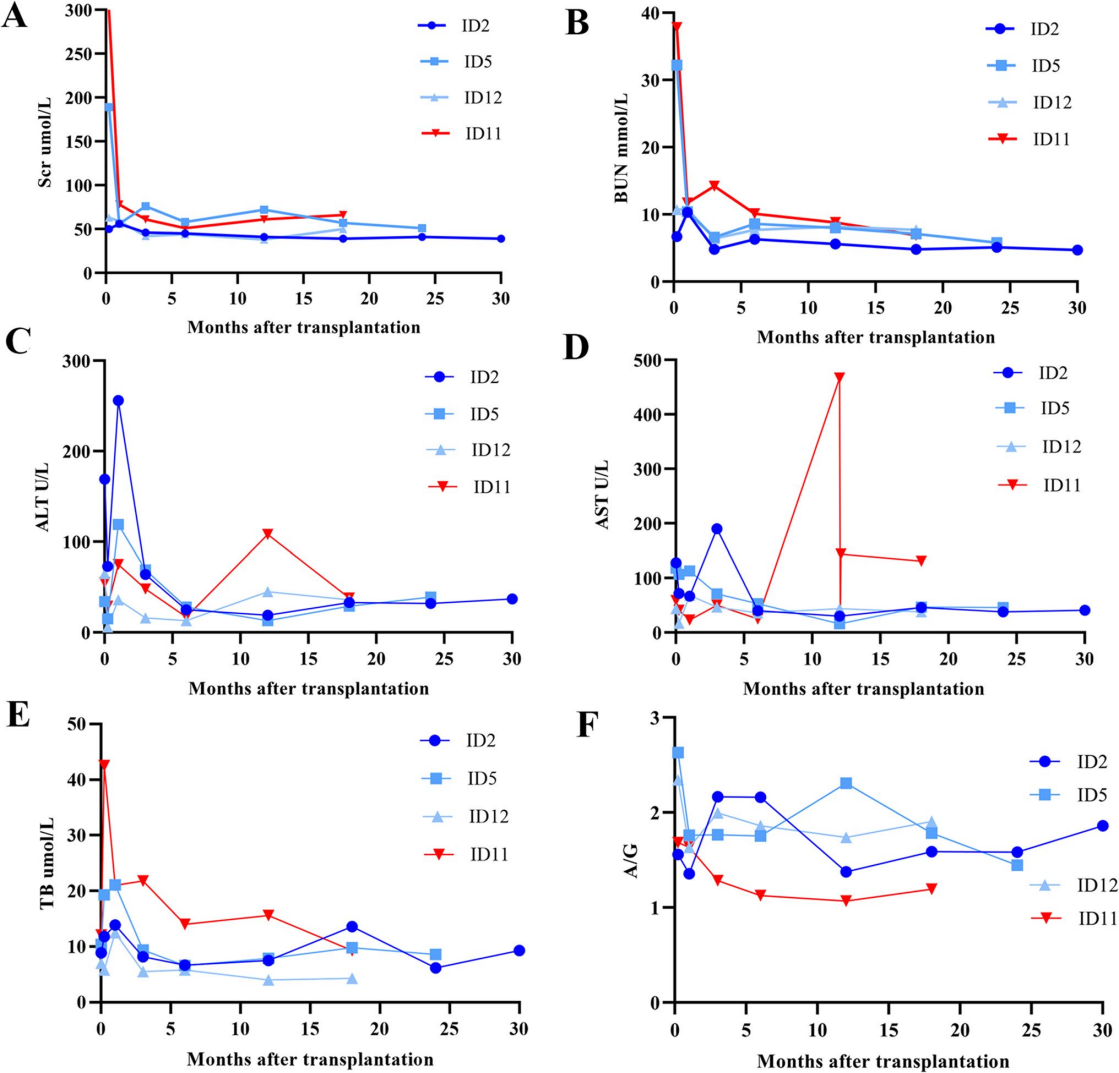
Follow-up of liver and kidney function after transplantation in children with Boichis syndrome. **A** Serum creatinine (Scr). **B** Blood urea nitrogen (BUN). **C** Alanine aminotransferase (ALT). **D** Aspartate aminotransferase (AST). **E** Total bilirubin (TB). **F** Albumin/globulin ratio (A/G)

The liver findings of the four children with Boichis syndrome are described in Table 4. One patient (number 11), who had portal hypertension, received CLKT. The patient had been followed-up for 18 months, and currently, blood indices (Scr, BUN, ALT, AST, TB, A/G) and other findings were relatively normal (Fig. 3A–F, red line). The other three children (numbers 2, 5, and 12) received kidney transplantation only.

**Table 4:** Phenotypic expression of liver disease in children with Boichis syndrome

Patient ID	Elevated liver enzymes	Hepatospleno-megaly	Enlarged hepatic parenchyma echo	Collapse of bile duct	Liver fibrosis	Portal hypertension
2	+	+	+	+	+	-
5	+	+	+	-	+	-
11	+	+	+	-	+	+
12	+	+	+	-	+	-

Considering the possibility of rapid development of hepatic fibrosis to portal hypertension after isolated kidney transplantation, the three children have been followed closely with liver function testing. Although kidney function recovered well in the three patients (Fig. 3A and B, blue lines), they developed symptoms of cholestasis in the early postoperative period, manifested by an increase in liver enzymes, bile acids, and total bilirubin. After treatment with ursodeoxycholic acid, blood indices (ALT, AST, TB, A/G) gradually returned to normal and overall tended to be stable (Fig. 3C–F, blue lines).

**Fig. 4:**
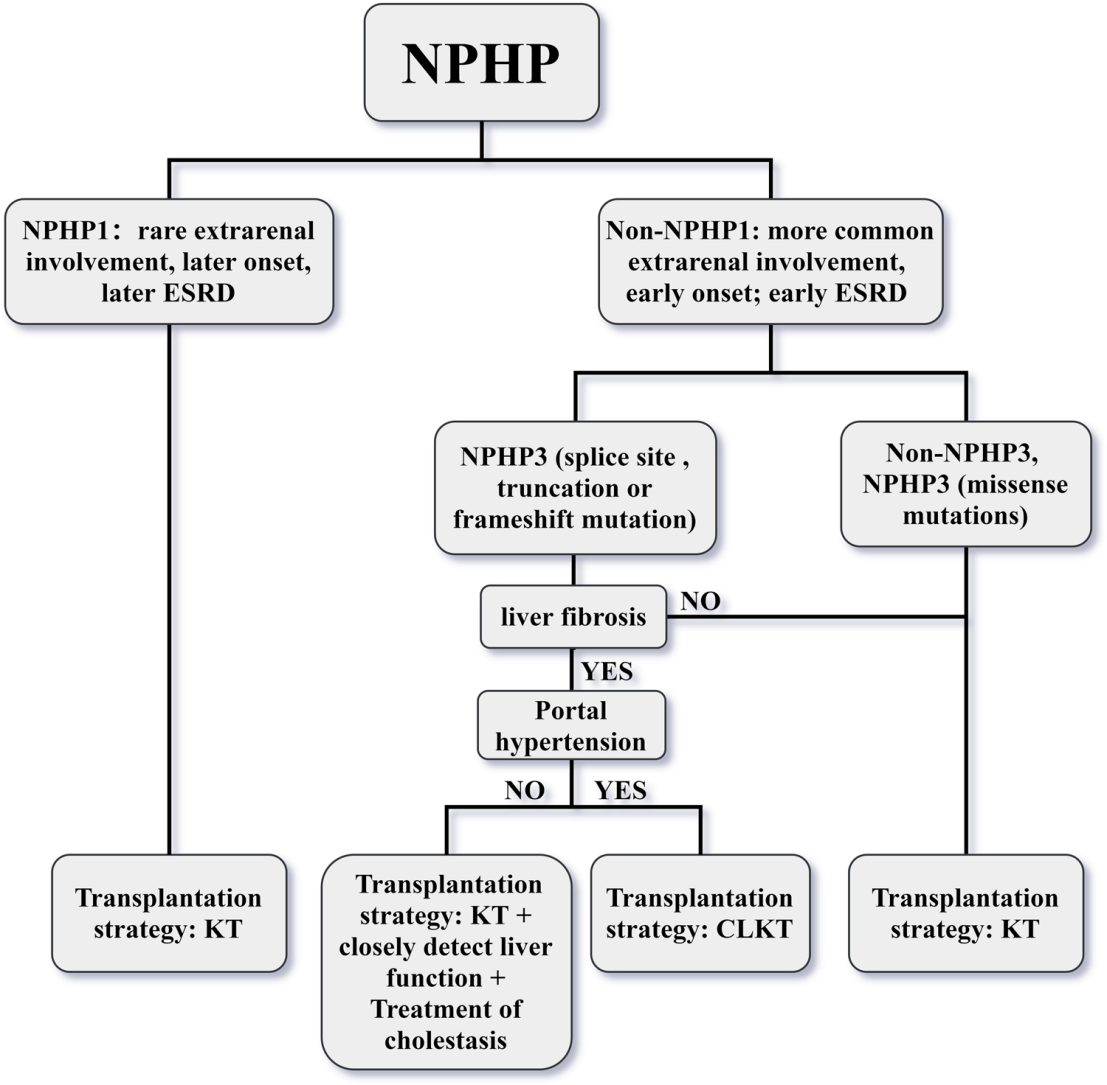
The summary of transplantation strategies for children with NPHP in this cohort. CLKT, combined liver and kidney transplantation; KT, kidney transplantation

A summary of transplantation strategies for children with NPHP is shown in Fig. 4.

## Discussion

In this study, we reviewed genotype and phenotype variations in children with NPHP, analyzed characteristics associated with *NPHP1* and non-*NPHP1* mutations with a focus on *NPHP3* pathogenic mutations, and summarized the treatment of patients with Boichis syndrome. We found that isolated kidney transplantation is feasible for patients with Boichis syndrome with mild to moderate liver fibrosis, while cholestasis can occur postoperatively and be treated symptomatically. Follow-up results after transplantation were acceptable.

Studies have shown that *NPHP1* is the most common gene to harbor pathogenic mutations [7, 22], and our results are consistent with those of the prior studies. Children with *NPHP1* pathogenic mutations reached kidney failure at a later age than those with non-*NPHP1* mutations. Additionally, most patients with *NPHP1* mutations exhibited isolated nephropathy and less extrarenal involvement. This suggests that pathogenic *NPHP1* mutations result in less severe phenotypes than other mutations. All patients in this cohort with *NPHP1* mutations showed isolated nephropathy, and patients with *NPHP1* mutations reported in the past had a probability of isolated nephropathy ranging from 76.5 to 90% [7, 8]. Although there were some differences, they all reflected the fact that *NPHP1* mutations rarely show extrarenal phenotypes.

While the frequency of non-*NPHP1* mutations was less than that of *NPHP1* mutations, non-*NPHP1* pathogenic mutations were associated with an earlier age of disease presentation and an earlier age of reaching kidney failure, as well as more common extrarenal involvement of the liver, eyes, and bones. In fact, in some cases, children have already progressed to kidney failure when they are first seen by a physician due to disease symptoms. Thus, differences between the age of disease presentation and age at reaching kidney failure in this study may not accurately reflect the inherent progression rate of NPHP. Of particular note, we found that *NPHP3* mutations are associated with a high frequency of liver involvement and other severe conditions. Previous studies have also observed that *NPHP3* mutations are associated with liver abnormalities [23].

Chaki et al. found 13 patients with *NPHP3* mutations in a total of 440 patients with NPHP, and a study by Tang et al. in China reported 15 patients with *NPHP3* mutations in 60 patients with NPHP [7, 8]. Chinese children with NPHP have a relatively high frequency of *NPHP3* mutations, which are characterized by rapid progression to kidney failure and liver involvement [7, 23]. Interestingly, children with liver involvement and kidney failure at an earlier age carry mutations that significantly impact protein function, including splice site mutations, truncation mutations, and frameshift mutations. Children with missense mutations in our study did not have extrarenal involvement, and the disease progressed slowly. Previous studies also support the notion that missense mutations in *NPHP3* are associated with mild NPHP, while loss of function mutations may result in a more severe phenotype [23, 24].

Otto et al. [25] reported that Boichis syndrome could manifest in patients with *TMEM67* mutations. We did not find any *TMEM67* mutations in the 29 patients included in our analysis, but the proportion of patients with *NPHP3* mutations characterized by Boichis syndrome was high. Treatment of Boichis syndrome that has reached kidney failure is still a topic for discussion. While a number of centers have reported the experience of treating Boichis syndrome [18, 20, 21], in general, the number of cases is small, and thus data regarding optimal management is insufficient. For children with Boichis syndrome, we chose the surgical management based on liver phenotype; one patient received CLKT and three kidney transplantation only. Overall, the postoperative follow-up results are satisfactory in all four patients.

Prior reports have suggested that portal hypertension is the direct or indirect cause of postoperative adverse events in patients with Boichis syndrome who received kidney transplantation alone [20, 21]. The survival rate and long-term prognosis of CLKT have improved in recent years. In CLKT, the transplanted liver has an immunoprotective effect on the transplanted kidney [26], and CLKT can avoid having to perform two separate major operations in a short time. Of our 29 patients, one patient (number 11) developed Boichis syndrome with portal hypertension, and thus we performed CLKT and follow-up results have been satisfactory. However, compared with single organ transplantation, CLKT is a more complicated procedure associated with greater surgical risk and trauma.

It is not necessary for all patients with Boichis syndrome to receive CLKT, so we performed isolated kidney transplantation in three patients with NPHP with mild or moderate liver fibrosis without portal hypertension and closely followed the recovery of liver function after the operation. We found that in the early postoperative period, these patients developed symptoms of cholestasis. Previous studies reported that *NPHP3* loss-of-function mutations could cause rapid worsening of liver fibrosis, but they did not indicate the cause of the rapid worsening. Cholestasis can cause chronic inflammation of the liver, leading to steatosis, a reduction of hepatic cells, diffuse fibrosis, and proliferation of blood vessels inside and outside the liver, with the subsequent gradual development of cirrhosis. Therefore, the rapid worsening of liver function after kidney transplantation might be related to the emergence of cholestasis, and timely treatment of cholestasis could prevent the rapid progression of liver damage. We treated cholestasis with ursodeoxycholic acid and found that timely treatment stabilized liver function and prevented worsening of liver function with good results. Thus, isolated kidney transplantation is feasible for patients with mild and moderate liver fibrosis, but without portal hypertension. However, it is necessary to pay close attention to postoperative changes in liver function and provide prompt treatment.

Of the 184 patients in this cohort, four developed post-transplant thrombosis, of whom three were diagnosed with NPHP. The rate of renal vascular thrombois was higher in NPHP patients (3/29, 10.3%) than non-NPHP patients (1/155, 0.65%) (*p* = 0.013). The mutated genes carried by patients with post-transplant embolism in our cohort were *WDR19*, *NEK8*, and *IQCB1*. Doreille et al. reported that thrombotic microangiopathy (TMA) was found in young adult NPHP patients with *TTC21B* and *WDR19* mutations [14]. This information may suggest a potential association between the genotype of NPHP and post-transplant thrombosis. There were five patients with *TTC21B* mutation and one patient was found to have TMA in the native kidney biopsy. However, no vascular thrombosis occurred in these five patients after transplantation. In addition, based on the findings during surgical exploration, we infer that vascular thrombosis initiated in the main renal vascular instead of microvascular circulation, and TMA was not reported in the pathological examination of removed allografts. Therefore, it is difficult to reasonably conclude that post-transplant thrombosis is related to these genes, while we believe this is a very noteworthy clinical phenomenon.

There are some shortcomings of this study that should be considered. The population studied in this cohort are all children with kidney failure, so the correlation between genotype and phenotype cannot be applied to children with NPHP before kidney failure. The postoperative follow-up time of children with Boichis syndrome was relatively short, with the longest 30 months, and the shortest only 18 months. Not all children with NPHP and liver involvement received a liver biopsy because of its invasive nature. Liver biopsy has not been performed to reassess liver fibrosis in patients with Boichis syndrome after isolated kidney transplantation, although the serum parameters of liver function maintain nearly normal.

## Conclusions

Overall, our results showed that different NPHP genotypes may have different phenotypes, and children with *NPHP3* mutations are more likely to develop Boichis syndrome. CLKT is necessary for patients with Boichis syndrome with portal hypertension, whereas isolated kidney transplantation is feasible for patients with Boichis syndrome with mild to moderate liver fibrosis and no portal hypertension. For children with Boichis syndrome, CLKT and isolated kidney transplantation have advantages and disadvantages. Choosing the surgical strategy based on phenotype may help to reduce the occurrence of postoperative adverse events.

## Supplementary Information

Below is the link to the electronic supplementary material.

Graphical Abstract (PPTX 115 KB)

Supplementary file2 (DOCX 17 KB)

Supplementary file3 (DOCX 25 KB)

Supplementary file4 (DOCX 44 KB)

## Data Availability

The data that support the findings of this study are available from the corresponding author on reasonable request.

## References

[1] Luo F, Tao YH (2018). Nephronophthisis: a review of genotype-phenotype correlation. Nephrology.

[2] Hildebrandt F, Attanasio M, Otto E (2009). Nephronophthisis: disease mechanisms of a ciliopathy. J Am Soc Nephrol.

[3] Heninger E, Otto E, Imm A, Caridi G, Hildebrandt F (2001). Improved strategy for molecular genetic diagnostics in juvenile nephronophthisis. Am J Kidney Dis.

[4] Gupta S, Ozimek-Kulik JE, Phillips JK (2021). Nephronophthisis-pathobiology and molecular pathogenesis of a rare kidney genetic disease. Genes (Basel).

[5] Simms RJ, Eley L, Sayer JA (2009). Nephronophthisis. Eur J Hum Genet.

[6] König J, Kranz B, König S, Schlingmann KP, Titieni A, Tönshoff B (2017). Phenotypic spectrum of children with nephronophthisis and related ciliopathies. Clin J Am Soc Nephro.

[7] Tang X, Liu C, Liu X, Chen J, Fan X, Liu J (2020). Phenotype and genotype spectra of a Chinese cohort with nephronophthisis-related ciliopathy. J Med Genet.

[8] Chaki M, Hoefele J, Allen SJ, Ramaswami G, Janssen S, Bergmann C (2011). Genotype-phenotype correlation in 440 patients with NPHP-related ciliopathies. Kidney Int.

[9] Braun DA, Hildebrandt F (2017). Ciliopathies. Cold Spring Harb Perspect Biol.

[10] Larrue R, Chamley P, Bardyn T, Lionet A, Gnemmi V, Cauffiez C (2020). Diagnostic utility of whole-genome sequencing for nephronophthisis. NPJ Genom Med.

[11] Srivastava S, Molinari E, Raman S, Sayer JA (2018). Many genes-one disease? Genetics of nephronophthisis (NPHP) and NPHP-associated disorders. Front Pediatr.

[12] Renkema KY, Giles RH, Lilien MR, Beales PL, Roepman R, Oud MM (2018). The KOUNCIL consortium: from genetic defects to therapeutic development for nephronophthisis. Front Pediatr.

[13] Tory K, Rousset-Rouviere C, Gubler MC, Moriniere V, Pawtowski A, Becker C (2009). Mutations of NPHP2 and NPHP3 in infantile nephronophthisis. Kidney Int.

[14] Doreille A, Raymond L, Lebre A, Linster C, Saraeva Lamri R, Karras A (2021). Nephronophthisis in young adults phenocopying thrombotic microangiopathy and severe nephrosclerosis. Clin J Am Soc Nephrol.

[15] Stokman MF, Saunier S, Benmerah A (2021). Renal ciliopathies: sorting out therapeutic approaches for nephronophthisis. Front Cell Dev Biol.

[16] Hamiwka LA, Midgley JP, Wade AW, Martz KL, Grisaru S (2008). Outcomes of kidney transplantation in children with nephronophthisis: an analysis of the North American Pediatric Renal Trials and Collaborative Studies (NAPRTCS) Registry. Pediatr Transplant.

[17] Boichis H, Passwell J, David R, Miller H (1973). Congenital hepatic fibrosis and nephronophthisis. Q J Med.

[18] Udagawa T, Kamei K, Ogura M, Tsutsumi A, Noda S, Kasahara M (2012). Sequential liver-kidney transplantation in a boy with congenital hepatic fibrosis and nephronophthisis from a living donor. Pediatr Transplant.

[19] Duclaux-Loras R, Bacchetta J, Berthiller J, Rivet C, Demède D, Javouhey E (2016). Pediatric combined liver–kidney transplantation: a single-center experience of 18 cases. Pediatr Nephrol.

[20] Zhang H, Luo J, Liu L, Li J, Fu Q, Chen W (2018). Transplantation for infantile nephronophthisis with loss-of-function mutation in NPHP3: lesson from a case. Pediatr Transplant.

[21] Tsukamoto T, Tanaka M, Komiya T, Ueda S, Takasu K, Takahara S (2008). Nephronophthisis complicated with hepatic fibrosis: an autopsy case with rupture of the splenic artery after renal transplantation. Clin Exp Nephrol.

[22] Halbritter J, Porath JD, Diaz KA, Braun DA, Kohl S, Chaki M (2013). Identification of 99 novel mutations in a worldwide cohort of 1,056 patients with a nephronophthisis-related ciliopathy. Hum Genet.

[23] Sun L, Tong H, Wang H, Yue Z, Liu T, Lin H (2016). High mutation rate of NPHP3 in 18 Chinese infantile nephronophthisis patients. Nephrology (Carlton).

[24] Bergmann C, Fliegauf M, Bruchle NO, Frank V, Olbrich H, Kirschner J (2008). Loss of nephrocystin-3 function can cause embryonic lethality, Meckel-Gruber-like syndrome, situs inversus, and renal-hepatic-pancreatic dysplasia. Am J Hum Genet.

[25] Otto EA, Tory K, Attanasio M, Zhou W, Chaki M, Paruchuri Y (2009). Hypomorphic mutations in meckelin (MKS3/TMEM67) cause nephronophthisis with liver fibrosis (NPHP11). J Med Genet.

[26] Ganschow R, Hoppe B (2015). Review of combined liver and kidney transplantation in children. Pediatr Transplant.

