# Supplementary material for: Genotype and phenotype analysis and transplantation strategy in children with kidney failure caused by NPHP
Source: Pediatr Nephrol. 2022 Oct 13;38(5):1609–20. doi: 10.1007/s00467-022-05763-3 (PMC10060285; doi:10.1007/s00467-022-05763-3)
Supplement: Supplementary file 1 — Graphical Abstract (PPTX 115 KB) [file 467_2022_5763_MOESM1_ESM.pptx]

## Slide 1
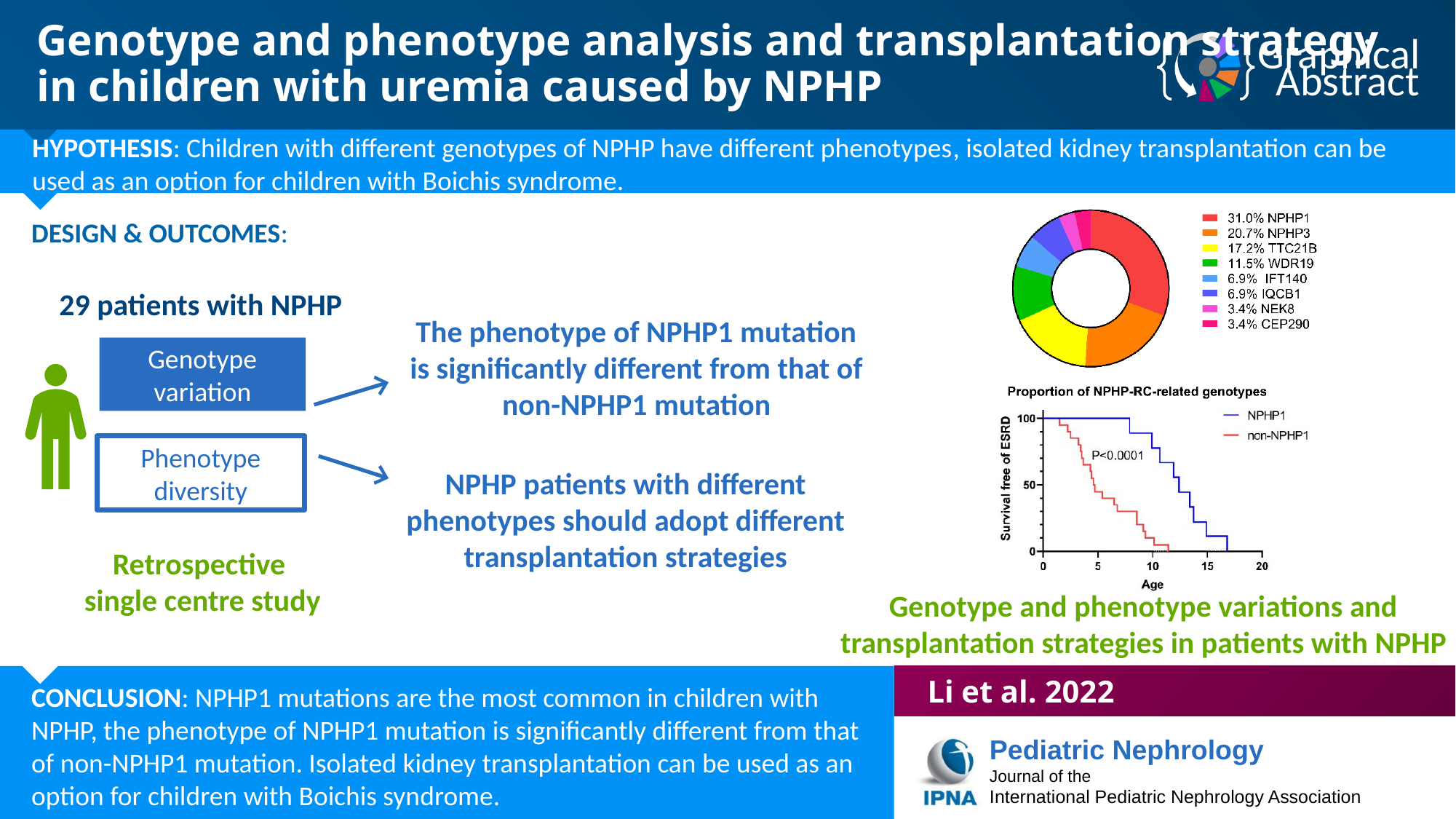

Genotype and phenotype analysis and transplantation strategy
in children with uremia caused by NPHP
HYPOTHESIS: Children with different genotypes of NPHP have different phenotypes, isolated kidney transplantation can be used as an option for children with Boichis syndrome.
DESIGN & OUTCOMES:
29 patients with NPHP
The phenotype of NPHP1 mutation is significantly different from that of non-NPHP1 mutation
Genotype variation
Phenotype diversity
NPHP patients with different phenotypes should adopt different transplantation strategies
Retrospective
single centre study
Genotype and phenotype variations and transplantation strategies in patients with NPHP
Li et al. 2022
CONCLUSION: NPHP1 mutations are the most common in children with NPHP, the phenotype of NPHP1 mutation is significantly different from that of non-NPHP1 mutation. Isolated kidney transplantation can be used as an option for children with Boichis syndrome.
