# Supplementary material for: Genotype and phenotype analysis and transplantation strategy in children with kidney failure caused by NPHP
Source: Pediatr Nephrol. 2022 Oct 13;38(5):1609–20. doi: 10.1007/s00467-022-05763-3 (PMC10060285; doi:10.1007/s00467-022-05763-3)
Supplement: Supplementary file 2 — Supplementary file2 (DOCX 17 KB) [file 467_2022_5763_MOESM2_ESM.docx]

Supplemental Table 1 List of genes contained in nephropathy gene panel

| *ACE* | *BBS4* | *DGKE* | *IFT172* | *NPHP1* | *SDCCAG8* | *TNXB* |
| --- | --- | --- | --- | --- | --- | --- |
| *ACTN4* | *BBS5* | *DIS3L2* | *IFT43* | *NPHP3* | *SIX1* | *TRIM32* |
| *ADCK4* | *BBS7* | *DMP1* | *IFT80* | *NPHP4* | *SIX5* | *TRPC6* |
| *AGT* | *BBS9* | *DYNC2H1* | *INF2* | *NPHS1* | *SLC12A1* | *TRPM6* |
| *AGTR1* | *BSND* | *EGF* | *INPP5E* | *NPHS2* | *SLC12A3* | *TTC21B* |
| *AGXT* | *CA2* | *ENPP1* | *INVS* | *OCRL* | *SLC1A1* | *TTC8* |
| *AHI1* | *CASR* | *EYA1* | *IQCB1* | *OFD1* | *SLC22A12* | *UMOD* |
| *ALMS1* | *CC2D2A* | *FGA* | *KAL1* | *PAX2* | *SLC2A2* | *UPK3A* |
| *ANKS6* | *CEP164* | *FGF23* | *KCNA1* | *PAX6* | *SLC34A1* | *WDPCP* |
| *APOA1* | *CEP290* | *FGFR1* | *KCNJ1* | *PDE6D* | *SLC3A1* | *WDR19* |
| *APRT* | *CEP41* | *FRAS1* | *KCNJ10* | *PHEX* | *SLC4A1* | *WDR34* |
| *AQP2* | *CLCN5* | *FREM2* | *KLHL3* | *PKD1* | *SLC4A4* | *WDR35* |
| *ARHGDIA* | *CLCNKB* | *FXYD2* | *LAMB2* | *PKD2* | *SLC5A2* | *WDR60* |
| *ARL6* | *CLDN16* | *GATA3* | *LMX1B* | *PKHD1* | *SLC7A7* | *WNK1* |
| *ATP6V0A4* | *CLDN19* | *GLA* | *LRIG2* | *PLCE1* | *SLC7A9* | *WNK4* |
| *ATP6V1B1* | *CNNM2* | *GLIS2* | *LYZ* | *PTPRO* | *SMARCAL1* | *WT1* |
| *AVPR2* | *COL4A3* | *GPC3* | *LZTFL1* | *REN* | *SOX17* | *XDH* |
| *B2M* | *COL4A4* | *GRHPR* | *MKKS* | *RET* | *TCTN2* | *ZNF423* |
| *B9D1* | *COL4A5* | *GRIP1* | *MKS1* | *ROBQ2* | *TCTN3* |  |
| *B9D2* | *COQ2* | *HNF1B* | *MUC1* | *RPGRIP1L* | *TMEM138* |  |
| *BBS1* | *COQ6* | *HOGA1* | *MYH9* | *SALL1* | *TMEM216* |  |
| *BBS10* | *CSPP1* | *HPSE2* | *MYO1E* | *SCNN1A* | *TMEM231* |  |
| *BBS12* | *CTNS* | *IFT122* | *NEK1* | *SCNN1B* | *TMEM237* |  |
| *BBS2* | *CUL3* | *IFT140* | *NEK8* | *SCNN1G* | *TMEM67* |  |
