# Supplementary material for: Genotype and phenotype analysis and transplantation strategy in children with kidney failure caused by NPHP
Source: Pediatr Nephrol. 2022 Oct 13;38(5):1609–20. doi: 10.1007/s00467-022-05763-3 (PMC10060285; doi:10.1007/s00467-022-05763-3)
Supplement: Supplementary file 3 — Supplementary file3 (DOCX 25 KB) [file 467_2022_5763_MOESM3_ESM.docx]

Supplemental Table 2 Disease-causing mutations information of 29 NPHP patients.

| **Patient** | **Mutation gene** | **Variants type** | **Nucleotide change** | **Amino acid change** | **ACMG classification** | **References of variants** | **hom/het** | **father** | **mather** |
| --- | --- | --- | --- | --- | --- | --- | --- | --- | --- |
| 1 | *WDR19* | missense variant | c.2963A>C | p.Gln988Pro | VUS | novel variant | het | Without this mutation | het |
| 1 | *WDR19* | frameshift variant | c.3967-3974del | p.Ala1324Glufs | VUS | novel variant | het | het | Without this variant |
| 2 | *NPHP3* | splicing variant | c.3697-2A>C | splice site | LP | PMID: 34031707 | het | Without this variant | het |
| 2 | *NPHP3* | missense variant | c.1181T>G | p.Ile394Ser | LP | novel variant | het | het | Without this variant |
| 3 | *TTC21B* | missense variant | c.T1552C | p.Cys518Arg | P | doi: 10.7499/j.issn.1008-8830.2019.06.015 | het | Without this variant | het |
| 3 | *TTC21B* | frameshift variant | c.530delA | p.Asp177fs | P | novel variant | het | het | Without this variant |
| 4 | *IQCB1* | nonsense variant | c.1090C>T | p. Arg 364* | LP | PMID: 23661368 | hom | het | het |
| 5 | *NPHP3* | splicing variant | c.2694-2_2694-1delAG | splice site | P | PMID: 20007846 | het | het | Without this variant |
| 5 | *NPHP3* | inframe variant | c.1304_1306delAAG | p.435del | P | PMID: 23559409 | het | Without this variant | het |
| 6 | *TTC21B* | splicing variant | c.2323-2A>G | splice site | P | novel variant | het | Without this variant | het |
| 6 | *TTC21B* | missense variant | c.379G>A | p.Ala127Thr | P | PMID: 27491411 | het | het | Without this variant |
| 7 | *NPHP3* | splicing variant | c.3813-3A>G | splice site | LP | PMID: 34295353 | hom | het | het |
| 8 | *WDR19* | missense variant | c.979T>C | p.Trp327 Arg | VUS | novel variant | het | het | Without this variant |
| 8 | *WDR19* | missense variant | c.3703G>A | p.Glu1235Lys | P | PMID: 35368817 | het | Without this variant | het |
| 9 | *CEP290* | splicing variant | c.2484-2A>G | splice site | P | PMID: 26047050 | het | het | Without this variant |
| 9 | *CEP290* | splicing variant | c.2586+1G>A | splice site | P | PMID: 26047050 | het | Without this variant | het |
| 10 | *NEK8* | missense variant | c.2018G>A | p.Cys673Tyr | VUS | novel variant | het | Without this variant | het |
| 10 | *NEK8* | missense variant | c.940G>A | p.Val314Met | VUS | novel variant | het | het | Without this variant |
| 11 | *NPHP3* | splicing variant | c.3813-3A>G | splice site | LP | PMID: 34295353 | het | het | Without this variant |
| 11 | *NPHP3* | missense variant | c.1135T>C | p.Cys379 Arg | VUS | PMID: 34295353 | het | Without this variant | het |
| 12 | *NPHP3* | nonsense variant | c.1817G>A | p.Trp606* | LP | PMID: 32173348 | het | Without this variant | het |
| 12 | *NPHP3* | inframe variant | c.1304_1306del | p.435del | P | PMID: 23559409 | het | het | Without this variant |
| 13 | *IFT140* | missense variant | c.745A>C | p.Thr249Pro | VUS | novel variant | het | Without this variant | het |
| 13 | *IFT140* | splicing variant | c.3141G>A | p.Lys1047 Lys | VUS | novel variant | het | het | Without this variant |
| 14 | *TTC21B* | missense variant | c.166G>A | p.Ala56Thr | VUS | novel variant | het | Without this variant | het |
| 14 | *TTC21B* | frameshift variant | c.1656_1659del | p.Cys552fs | P | PMID: 21258341 | het | het | Without this variant |
| 15 | *TTC21B* | nonsense variant | c.264_267dupTAGA | p.Glu90* | P | PMID: 23559409 | het | Without this variant | het |
| 15 | *TTC21B* | missense variant | c.380C>T | p.Ala127Val | LP | PMID: 32173348 | het | het | Without this variant |
| 16 | *IQCB1* | nonsense variant | c.1504C>T | p.Arg502* | P | PMID: 25851290 | het | het | Without this variant |
| 16 | *IQCB1* | frameshift variant | c.1342_1343insC | p.Gln448fs | P | novel variant | het | Without this variant | het |
| 17 | *WDR19* | nonsense variant | c.641T>A | p.Leu214* | LP | PMID: 34295353 | het | Without this variant | het |
| 17 | *WDR19* | missense variant | c.2579C>A | p.Ala860Asp | VUS | novel variant | het | het | Without this variant |
| 18 | *IFT140* | missense variant | c.3712G>A | p.Ala1238Thr | VUS | novel variant | het | Without this variant | het |
| 18 | *IFT140* | missense variant | c.2101G>A | p.Glu701Lys | VUS | novel variant | het | het | Without this variant |
| 19 | *TTC21B* | frameshift variant | c.1656-1659del | p.Cys552fs | P | PMID: 21258341 | het | Without this variant | het |
| 19 | *TTC21B* | missense variant | c.1552T>C | P.Cys518 Arg | LP | PMID: 28124483 | het | het | Without this variant |
| 20 | *NPHP3* | missense variant | c.2132A>G | p.Asn711Ser | VUS | PMID: 26184788 | het | Without this variant | het |
| 20 | *NPHP3* | missense variant | c.1082C>G | p.Ser361Cys | LP | PMID: 34295353 | het | het | Without this variant |
| 21 | *NPHP1* | frameshift variant | c.1298delA | p.Lys433fs | P | PMID: 31523374,32173348 | het | het | unknown |
| 21 | *NPHP1* | gene deletion | Deletion of exon1-20 | N/A | P | PMID: 15138899,15689444 | het | Without this variant | unknown |
| 22 | *NPHP1* | gene deletion | Homozygous deletion of exon1-20 | N/A | P | PMID: 15138899,15689444 | hom | het | het |
| 23 | *NPHP1* | gene deletion | Homozygous deletion of exon1-20 | N/A | P | PMID: 15138899,15689444 | hom | het | het |
| 24 | *NPHP1* | gene deletion | Homozygous deletion of exon1-20 | N/A | P | PMID: 15138899,15689444 | hom | het | het |
| 25 | *NPHP1* | gene deletion | Homozygous deletion of exon1-20 | N/A | P | PMID: 15138899,15689444 | hom | het | het |
| 26 | *NPHP1* | gene deletion | Homozygous deletion of exon1-20 | N/A | P | PMID: 15138899,15689444 | hom | het | het |
| 27 | *NPHP1* | gene deletion | Homozygous deletion of exon1-20 | N/A | P | PMID: 15138899,15689444 | hom | unknown | unknown |
| 28 | *NPHP1* | gene deletion | Homozygous deletion of exon1-20 | N/A | P | PMID: 15138899,15689444 | hom | het | het |
| 29 | *NPHP1* | gene deletion | Homozygous deletion of exon1-20 | N/A | P | PMID: 15138899,15689444 | hom | unknown | unknown |
