# Supplementary material for: Genotype and phenotype analysis and transplantation strategy in children with kidney failure caused by NPHP
Source: Pediatr Nephrol. 2022 Oct 13;38(5):1609–20. doi: 10.1007/s00467-022-05763-3 (PMC10060285; doi:10.1007/s00467-022-05763-3)
Supplement: Supplementary file 4 — Supplementary file4 (DOCX 44 KB) [file 467_2022_5763_MOESM4_ESM.docx]

STROBE Statement—checklist of items that should be included in reports of observational studies

|  | Item No. | Recommendation | Page  No. | Relevant text from manuscript |
| --- | --- | --- | --- | --- |
| **Title and abstract** | 1 | (*a*) Indicate the study’s design with a commonly used term in the title or the abstract | Page 3 | The records of children with NPHP treated at our center from 01/2018 to 03/2021 were retrospectively reviewed. Inclusion criteria were a diagnosis of NPHP, received kidney transplantation, and received whole exome sequencing (WES) or nephropathy gene panel testing. |
|  |  | (*b*) Provide in the abstract an informative and balanced summary of what was done and what was found | Page 3 | NPHP1 mutations are the most common in children with NPHP, and the phenotype of NPHP1 mutation is significantly different from that of non-NPHP1 mutation. For NPHP patients with mild to moderate liver fibrosis without portal hypertension, timely treatment of cholestasis could prevent the rapid progress of liver function damage after isolated kidney transplantation. |
| Introduction | | | |  |
| Background/rationale | 2 | Explain the scientific background and rationale for the investigation being reported | Page 5/6 | As nephropathy progresses to the kidney failure stage, the involvement of extrarenal organs also progresses. Thus, the analysis of the phenotype of uremic patients is helpful for the perioperative management of kidney or other organ transplantation.  However, there is no clear guidance on the most optimal surgical strategy for patients with mild to moderate liver fibrosis (especially those with high-risk genotypes). CLKT can solve both liver fibrosis and kidney failure, yet the surgical risk is high. The surgical success rate of isolated kidney transplantation is high; however, it is uncertain when liver fibrosis progressed to the indication of liver transplantation. |
| Objectives | 3 | State specific objectives, including any prespecified hypotheses | Page 6 | Thus, the purpose of this study was to examine the association between NPHP genotype and phenotype in children to provide references for clinicians when determining an appropriate transplantation strategy. |
| Methods | | | |  |
| Study design | 4 | Present key elements of study design early in the paper | Page 6 | The genotype and phenotype of children with NPHP-RC treated at our center from January 1, 2018 to March 31, 2021 were retrospectively reviewed. |
| Setting | 5 | Describe the setting, locations, and relevant dates, including periods of recruitment, exposure, follow-up, and data collection | Page 6 | The genotype and phenotype of children with NPHP-RC treated at our center from January 1, 2018 to March 31, 2021 were retrospectively reviewed.  Data extracted from the medical records included medical and disease history, including age at presentation of NPHP-RC and age when kidney failure was reached. Laboratory data extracted included serum creatinine (Scr), blood urea nitrogen (BUN), albumin (ALB), globulin (GLB), alanine aminotransferase (ALT), aspartate aminotransferase (AST), and total bilirubin (TB) levels. All children underwent abdominal ultrasound. A liver biopsy was performed if abdominal ultrasound indicated liver fibrosis when the informed consent was available. All the children underwent chest, extremity, and pelvis x-rays. All children received visual acuity and visual field evaluation, slit lamp examination if necessary, and optical coherence tomography and electrophysiological examinations in cooperative patients suspected of having retinitis pigmentosa. Each child underwent an echocardiogram to check for structural defects in the heart. Head MRI examinations were performed in children with neurological abnormalities found by physical examination to detect cerebellar vermis hypoplasia and other structural brain abnormalities. All patients were followed up through outpatient clinic and telephone after renal transplantation. Postoperative eGFR, graft survival, patient survival, rejection, and recurrence of primary disease were obtained by follow-up. |
| Participants | 6 | (*a*) *Cohort study*—Give the eligibility criteria, and the sources and methods of selection of participants. Describe methods of follow-up  *Case-control study*—Give the eligibility criteria, and the sources and methods of case ascertainment and control selection. Give the rationale for the choice of cases and controls  *Cross-sectional study*—Give the eligibility criteria, and the sources and methods of selection of participants | Page 6/7 | Inclusion criteria were a diagnosis of NPHP, received kidney or combined liver and kidney transplantation (CLKT), received whole exome sequencing (WES) or nephropathy gene panel testing and were found to have a pathogenic gene mutation associated with NPHP, had complete medical records, and were ≤ 18 years of age. Diagnosis of NPHP was based on the clinical diagnostic criteria for NPHP proposed by Chaki et al. Exclusion criteria were: 1) Whole exome sequencing (WES) or a nephropathy gene panel study was not performed; 2) A NPHP-related pathogenic mutation was not present; 3) The patient did not meet the clinical diagnostic criteria of a NPHP-RC.  All patients were followed up through outpatient clinic and telephone after renal transplantation. |
|  |  | (*b*) *Cohort study*—For matched studies, give matching criteria and number of exposed and unexposed  *Case-control study*—For matched studies, give matching criteria and the number of controls per case |  | N/A |
| Variables | 7 | Clearly define all outcomes, exposures, predictors, potential confounders, and effect modifiers. Give diagnostic criteria, if applicable | Page 6/7 | Phenotypic screening mainly comes from clinical examination.  Postoperative eGFR, graft survival, patient survival, rejection and recurrence of primary disease were obtained by follow-up.  Diagnosis of NPHP was based on the clinical diagnostic criteria for NPHP proposed by Chaki et al. |
| Data sources/ measurement | 8* | For each variable of interest, give sources of data and details of methods of assessment (measurement). Describe comparability of assessment methods if there is more than one group | Page 7/8 | Data extracted from the medical records included medical and disease history, including age at presentation of NPHP-RC and age when kidney failure was reached. Laboratory data extracted included serum creatinine (Scr), blood urea nitrogen (BUN), albumin (ALB), globulin (GLB), alanine aminotransferase (ALT), aspartate aminotransferase (AST), and total bilirubin (TB) levels.  All children underwent abdominal ultrasound. A liver biopsy was performed if abdominal ultrasound indicated liver fibrosis when the informed consent was available. All the children underwent chest, extremity, and pelvis x-rays. All children received visual acuity and visual field evaluation, slit lamp examination if necessary, and optical coherence tomography and electrophysiological examinations in cooperative patients suspected of having retinitis pigmentosa. Each child underwent an echocardiogram to check for structural defects in the heart. Head MRI examinations were performed in children with neurological abnormalities found by physical examination to detect cerebellar vermis hypoplasia and other structural brain abnormalities.  WES was performed using MyGentics and Wuxi NextCODE. Genomic DNA was extracted from blood lymphocytes. Exon capture was performed using Agilent SureSelect Human all Exome V5 Kit, NimbleGen, or MyGenostics Gencap Capture techniques, and then NGS was performed on the Illumina HighSeq sequencing platform. At least 98% of the target sequences were sequenced at a 20× reading depth.  After removing reads containing adaptor sequences and low-quality reads, clean data were mapped to human reference genome assembly (NCBI build 37/hg19) using the Burrow-Wheeler Aligner (BWA). Single nucleotide polymorphisms (SNP) and small insertions and deletions (INDELs) were detected using a Genome Analysis Toolkit (GATK). Allele frequency was determined by annotating the variations using public databases (genomAD, HGMD, and the 1000 Genomes Project). Changes that represented synonymous and common variants (minor allele frequency > 1%) were discarded. SNP variant deleteriousness was predicted by SIFT, Polyhen2, and Mutation Taster. Mutation screening was prioritized based on known disease-causing genes and nephropathy-associated genes. Evidence of disease causality was assessed using ClinVar and the Human Genome Mutation Database (HGMD), followed by a manual review of the cited primary literature. Copy number variation (CNV) analysis was performed using WES data, and then validated by PCR. Using the ALB gene as the internal reference gene, the copy number of exon 1-20 of NPHP1 was detected by fluorescence quantitative PCR with normal control samples and proband and family samples.  The nephropathy gene panel includes 162 genes causative or associated with nephropathy, as well as genes that may cause phenocopies in humans or related phenotypes in animal models (Supplementary Table S1). A custom NimbleGen SeqCap EZ Choice Library (NimbleGen; Roche, Madison, WI) was used to capture all exons and exon-intron boundaries (plus 50 base pairs at each end) of these genes for a final targeted region of 1.05 Mb. |
| Bias | 9 | Describe any efforts to address potential sources of bias | Page 8 | Pathogenicity assessment was conducted by a team of nephrologists and molecular geneticists. According to American College of Medical Genetics (ACMG) guidelines, mutations in known pathogenic genes whose pathogenicity levels are "pathogenic", " likely pathogenic " and " variant of unknown significance (VUS)" are defined as diagnostic variants. All diagnostic variants were confirmed by Sanger sequencing with segregation. |
| Study size | 10 | Explain how the study size was arrived at | Page 6/7 | The genotype and phenotype of children with NPHP-RC treated at our center from January 1, 2018 to March 31, 2021 were retrospectively reviewed. Inclusion criteria were a diagnosis of NPHP, received kidney or combined liver and kidney transplantation (CLKT), received whole exome sequencing (WES) or nephropathy gene panel testing and were found to have a pathogenic gene mutation associated with NPHP, had complete medical records, and were ≤ 18 years of age. Diagnosis of NPHP was based on the clinical diagnostic criteria for NPHP proposed by Chaki et al. |

Continued on next page

| Quantitative variables | 11 | Explain how quantitative variables were handled in the analyses. If applicable, describe which groupings were chosen and why | Page 7 | Continuous data were expressed as mean ± standard deviation or median and interquartile range (IQR), and categorical data as count (percentage). |
| --- | --- | --- | --- | --- |
| Statistical methods | 12 | (*a*) Describe all statistical methods, including those used to control for confounding | Page 8/9 | Comparisons of patients with and without NPHP1 mutations was performed using the t test. Kidney survival analysis between children with NPHP1 mutations and those with non-NPHP1 mutations was performed by Kaplan-Meier analysis. |
|  |  | (*b*) Describe any methods used to examine subgroups and interactions |  | N/A |
|  |  | (*c*) Explain how missing data were addressed |  | N/A |
|  |  | (*d*) *Cohort study*—If applicable, explain how loss to follow-up was addressed  *Case-control study*—If applicable, explain how matching of cases and controls was addressed  *Cross-sectional study*—If applicable, describe analytical methods taking account of sampling strategy |  | N/A |
|  |  | (*e*) Describe any sensitivity analyses |  | N/A |
| Results | | | | |
| Participants | 13* | (a) Report numbers of individuals at each stage of study—eg numbers potentially eligible, examined for eligibility, confirmed eligible, included in the study, completing follow-up, and analysed | Page 9 | The records of 184 children and adolescents who received kidney transplantation at our center were identified and reviewed from January 1, 2018, to March 31, 2021. Of the 184 patients, 106 received WES or nephropathy gene panel analysis due to suspected hereditary nephropathy; therefore, the 78 patients who did not receive these tests were excluded. The clinical diagnosis of 33 children tended to NPHP and carried variants in genes related to NPHP, but four children were excluded because they carried monoallelic variants. Twenty-nine children (17 boys, 12 girls) were eventually included in the analysis. |
|  |  | (b) Give reasons for non-participation at each stage | Page 9 | therefore, the 78 patients who did not receive these tests were excluded.  four children were excluded because they carried monoallelic variants. |
|  |  | (c) Consider use of a flow diagram | Page 9 | Fig1 |
| Descriptive data | 14* | (a) Give characteristics of study participants (eg demographic, clinical, social) and information on exposures and potential confounders | Page 9 | The clinical data of the 29 children is summarized in Table 1. The mean age of NPHP presentation was 7.1 ± 3.81 years, and the mean age of reaching kidney failure was 7.8 ± 4.23 years. The median follow-up time was 21 months (IQR: 10-27 months). The median eGFR value post-transplant is 100 ml/min/1.73m2 (IQR: 91-112). One child underwent CLKT, and the others received kidney transplantation only. |
|  |  | (b) Indicate number of participants with missing data for each variable of interest |  | N/A |
|  |  | (c) *Cohort study*—Summarise follow-up time (eg, average and total amount) | Page 9 | The median follow-up time was 21 months (IQR: 10-27 months). |
| Outcome data | 15* | *Cohort study*—Report numbers of outcome events or summary measures over time | Page 10 | One child underwent CLKT, and the others received kidney transplantation only. Graft loss occurred in 3 patients due to acute thrombosis or acute kidney vein torsion. All 3 patients received a second transplantation; 2 grafts survived well after the second transplant and in one case there was secondary acute thrombosis. There was no rejection or recurrence of primary disease, and there was no recipient death.  In all patients, mutations in NPHP1 (n = 9, 31%) were the most common genetic defects. Non-NPHP1 mutations included mutations in NPHP3 (n = 6, 20.7%), TTC21B (n = 5, 17.2%), WDR19 (n = 3), IFT140 (n = 2), IQCB1 (n = 2), NEK8 (n = 1), and CEP290 (n = 1).  With respect to mutation types, NPHP1 mutations are quite different from non-NPHP1 mutations. Of the 9 children who carried NPHP1 pathogenic mutations, 8 had homozygous full-gene deletions of NPHP1. One patient with a NPHP1 pathogenic mutation had a 1 allele point mutation, which led to a frameshift mutation, and the other allele had a large fragment deletion. Of all the non-NPHP1 mutations, only 2 patients had homozygous mutations and the other 18 patients had compound heterozygous mutations. A total of 38 pathogenic mutations were found in the non-NPHP1 mutation group, including 5 nonsense variants (13.2%), 5 frameshift variants (13.2%), 2 inframe variants (5.3%), 8 splicing variants (21.1%) and 18 missense variants (47.4%) |
|  |  | *Case-control study—*Report numbers in each exposure category, or summary measures of exposure |  | N/A |
|  |  | *Cross-sectional study—*Report numbers of outcome events or summary measures |  | N/A |
| Main results | 16 | (*a*) Give unadjusted estimates and, if applicable, confounder-adjusted estimates and their precision (eg, 95% confidence interval). Make clear which confounders were adjusted for and why they were included |  | N/A |
|  |  | (*b*) Report category boundaries when continuous variables were categorized |  | N/A |
|  |  | (*c*) If relevant, consider translating estimates of relative risk into absolute risk for a meaningful time period |  | N/A |

Continued on next page

| Other analyses | 17 | Report other analyses done—eg analyses of subgroups and interactions, and sensitivity analyses |  | N/A |
| --- | --- | --- | --- | --- |
| Discussion | | | | |
| Key results | 18 | Summarise key results with reference to study objectives | Page 12/13 | In this study, we reviewed genotype and phenotype variations in children with NPHP, analyzed characteristics associated with NPHP1 and non-NPHP1 mutations with a focus on NPHP3 pathogenic mutations, and summarized the treatment of patients with Boichis syndrome. We found that isolated kidney transplantation is feasible for patients with Boichis syndrome who have mild to moderate liver fibrosis, while cholestasis can occur postoperative and can be treated symptomatically. Follow-up results after transplantation were acceptable. |
| Limitations | 19 | Discuss limitations of the study, taking into account sources of potential bias or imprecision. Discuss both direction and magnitude of any potential bias | Page 16 | There are some shortcomings of this study that should be considered. The population studied in this cohort are all children with kidney failure, so the correlation between genotype and phenotype cannot be applied to children with NPHP before kidney failure. The postoperative follow-up time of children with Boichis syndrome was relatively short, with the longest 30 months, and the shortest only 18 months. Not all children with NPHP and liver involvement received a liver biopsy because of its invasive nature. Liver biopsy has not been performed to reassess liver fibrosis in patients with Boichis syndrome after isolated kidney transplantation, although the serum parameters of liver function maintain nearly normal. |
| Interpretation | 20 | Give a cautious overall interpretation of results considering objectives, limitations, multiplicity of analyses, results from similar studies, and other relevant evidence | Page 13-16 | Studies have shown that NPHP1 is the most common gene to harbor pathogenic mutations [7, 22], and our results are consistent with those of the prior studies. Children with NPHP1 pathogenic mutations reached kidney failure at a later age than those with non-NPHP1 mutations. Additionally, most patients with NPHP1 mutations exhibited isolated nephropathy and less extrarenal involvement. This suggests that pathogenic NPHP1 mutations result in less severe phenotypes than other mutations. All patients in this cohort with NPHP1 mutations showed isolated nephropathy, and patients with NPHP1 mutations reported in the past had a probability of isolated nephropathy ranging from 76.5% to 90% [7-8]. Although there were some differences, they all reflected the fact that NPHP1 mutations rarely showed extrarenal phenotypes.  Of particular note, we found that NPHP3 mutations are associated with a high frequency of liver involvement and other severe conditions. Previous studies have also observed that NPHP3 mutations are associated with liver abnormalities [23].  Chaki et al. found 13 patients with NPHP3 mutations in a total of 440 patients with NPHP, and a study by Tang et al. in China reported 15 patients with NPHP3 mutations in 60 patients with NPHP [7-8]. Chinese children with NPHP have a relatively high frequency of NPHP3 mutations, which are characterized by rapid progression to kidney failure and liver involvement [7, 23]. |
| Generalisability | 21 | Discuss the generalisability (external validity) of the study results | Page 13-16 | All patients in this cohort with NPHP1 mutations showed isolated nephropathy, and patients with NPHP1 mutations reported in the past had a probability of isolated nephropathy ranging from 76.5% to 90%. Although there were some differences, they all reflected the fact that NPHP1 mutations rarely showed extrarenal phenotypes. Previous studies have also observed that NPHP3 mutations are associated with liver abnormalities. Previous studies also support that the missense mutations in NPHP3 are associated with mild NPHP, while loss of function mutations may result in a more severe phenotype.  Prior reports have suggested that portal hypertension is the direct or indirect cause of postoperative adverse events in patients with Boichis syndrome who received kidney transplantation alone [20-21]. The survival rate and long-term prognosis of CLKT have improved in recent years. In CLKT, the transplanted liver has an immunoprotective effect on the transplanted kidney [26], and CLKT can avoid having to perform 2 separate major operations in a short time. In our 29 patients, 1 patient (number 11) developed Boichis syndrome with portal hypertension and thus we performed CLKT and follow-up results have been satisfactory. However, compared with single organ transplantation CLKT is a more difficult procedure associated with greater surgical risk and surgical trauma. It is not necessary for all patients with Boichis syndrome to receive CLKT, so we performed isolated kidney transplantation in 3 NPHP patients with mild or moderate liver fibrosis without portal hypertension and closely followed the recovery of liver function after the operation. We found that in the early postoperative period these patients developed symptoms of cholestasis. Previous studies reported that NPHP3 loss-of-function mutations can cause rapid worsening of liver fibrosis, but they did not indicate the cause of the rapid worsening. Cholestasis can cause chronic inflammation of the liver, leading to steatosis, a reduction of hepatic cells, diffuse fibrosis, and proliferation of blood vessels inside and outside the liver, with the subsequent gradual development of cirrhosis. Therefore, the rapid worsening of liver function after kidney transplantation might be related to the emergence of cholestasis, and timely treatment of cholestasis could prevent the rapid progression of liver damage. We treated cholestasis with ursodeoxycholic acid and found that timely treatment stabilized liver function and prevented worsening of liver function with good results. Thus, isolated kidney transplantation is feasible for NPHP patients with mild and moderate liver fibrosis, but without portal hypertension. However, it is necessary to pay close attention to postoperative changes of liver function and provide prompt treatment. |
| Other information | |  | | |
| Funding | 22 | Give the source of funding and the role of the funders for the present study and, if applicable, for the original study on which the present article is based | Page 17 | This study was supported by Science and Technology Planning Project of Guangdong Province, China (2015B020226002, 2017A020215012), National Natural Science Foundation of China (81870511, 82170770, 31800758), Key Scientific and Technological Program of Guangzhou City (201803040011, 201903010058), Guangdong Provincial Key Laboratory on Organ Donation and Transplant Immunology (2017B030314018, 2020B1212060026), and Guangdong Provincial International Cooperation Base of Science and Technology (Organ Transplantation, 2020A0505020003). |

*Give information separately for cases and controls in case-control studies and, if applicable, for exposed and unexposed groups in cohort and cross-sectional studies.

**Note:** An Explanation and Elaboration article discusses each checklist item and gives methodological background and published examples of transparent reporting. The STROBE checklist is best used in conjunction with this article (freely available on the Web sites of PLoS Medicine at http://www.plosmedicine.org/, Annals of Internal Medicine at http://www.annals.org/, and Epidemiology at http://www.epidem.com/). Information on the STROBE Initiative is available at www.strobe-statement.org.
